# A study of ghrelin and leptin levels and their relationship to metabolic profiles in obese and lean Saudi women with polycystic ovary syndrome (PCOS)

**DOI:** 10.1186/s12944-018-0839-9

**Published:** 2018-08-21

**Authors:** Maha H. Daghestani, Mazin Daghestani, Mamoon Daghistani, Akmal El-Mazny, Geir Bjørklund, Salvatore Chirumbolo, Samar H. Al Saggaf, Arjumand Warsy

**Affiliations:** 1 Department of Zoology, King Saud University, Riyadh, Saudi Arabia; 2 Department of Obstetrics and Gynecology, Umm Al-Qura University, Makkah, Saudi Arabia; 3 Department of Surgery, King Abdulaziz Medical City, National Guard Health Affairs, Jeddah, Saudi Arabia; 4 Department of Obstetrics and Gynecology, College of Medicine, University of Cairo, Kasr Al-ainy, Cairo, Egypt; 5 Council for Nutritional and Environmental Medicine, Mo i Rana, Norway; 6 Department of Neurological and Movement Sciences, University of Verona, Verona, Italy; 7 Department of Anatomy, College of Medicine, King Abdulaziz University, Jeddah, Saudi Arabia; 8 Central Laboratory, Female Center for Scientific and Medical Colleges, King Saud University, Riyadh, Saudi Arabia

**Keywords:** Polycystic ovary syndrome, Ghrelin, Leptin, Insulin, insulin resistance

## Abstract

**Background:**

Polycystic ovary syndrome (PCOS) is considered as one of the most frequently encountered hormonal pathologies in women during their reproductive years. Leptin and ghrelin, peptide hormones with adipostatic and orexigenic effect, respectively, seem to be involved in the metabolic changes that occur in PCOS. The aim of this study was to determine serum ghrelin and leptin levels in obese and lean Saudi women with PCOS and to investigate their relationship to the metabolic profiles in these women.

**Methods:**

This study was conducted as a prospective, observational, cross-sectional, case-control study, at the Department of Obstetrics and Gynecology, Al-Noor Hospital, Makkah, Kingdom of Saudi Arabia. The study population included 252 women [130 women with PCOS (diagnosed according to the Rotterdam ESHRE/ASRM-Sponsored PCOS Consensus, 2003) and 122 normo-ovulatory women as matched controls] attending the outpatient Gynecology Clinic. Demographic details were recorded, blood was extracted following overnight fast and serum was used for the determination of serum ghrelin and leptin levels and other hormonal and biochemical parameters including total cholesterol, triglycerides, high-density lipoprotein (HDL) cholesterol, low-density lipoprotein (LDL) cholesterol, glucose, and insulin. Insulin resistance and sensitivity were calculated as HOMA-IR and HOMA-S.

**Results:**

No significant differences in ghrelin (*P* = 0.1830) and leptin (*P* = 0.8329) levels were detected between the PCOS and control groups. However, ghrelin levels were significantly lower; and leptin levels were significantly higher in obese PCOS patients in comparison with lean patients (*P* = 0.0001 for both). In the PCOS group, there were significant correlations between ghrelin and leptin levels with Body Mass Index (BMI), waist-hip ratio, total cholesterol, triglycerides, HDL, LDL and insulin levels. Multiple regression analysis demonstrated that insulin was the main determinant for ghrelin (*R*^*2*^ = 0.316) and leptin (*R*^*2*^ = 0.352) levels (*P* = 0.0001 for both).

**Conclusions:**

Although serum ghrelin and leptin levels were found to be normal in women with PCOS; yet, there is a relationship, possibly linked to obesity, hyperinsulinemia and insulin resistance between these levels and metabolic profile of Saudi PCOS.

**Electronic supplementary material:**

The online version of this article (10.1186/s12944-018-0839-9) contains supplementary material, which is available to authorized users.

## Background

In 1935, Stein and Leventhal first described polycystic ovary syndrome (PCOS) in seven women suffering from amenorrhea, enlarged ovaries with multiple cysts, and hirsutism [1]. Currently, PCOS is considered as one of the most frequently encountered hormonal pathologies in women during their reproductive years, occurring in most populations of the World [2, 3] and prevalence as high as 15–20% has been reported in some studies [4]. It is a heterogeneous complex genetic trait of multifactorial nature where both genetic and environmental factors contribute to the underlying pathophysiological mechanisms [5]. It has drawn significant attention as it is considered as a major cause of anovulatory infertility in women of childbearing age, and is the cause of several other complications including endocrine, metabolic, hemostatic and hepatic derangements [6, 7]. These recent studies have highlighted the association between PCOS and metabolic syndrome, obesity, insulin resistance, cardiovascular diseases and liver diseases including cirrhosis, liver tumors and fatty liver [6, 8].

Obesity is considered as one of the major factor predisposing to the development of PCOS, since 35-80% of the women suffering from PCOS are reported to be overweight or obese [9–12]. However, it is not the only factor, since many PCOS patients are lean [13, 14]. Type 1 and Type 2 diabetes mellitus, and gestational diabetes have been associated with an increased prevalence of PCOS [15]. Some studies have implicated leptin and ghrelin as possible factors contributing to the development of PCOS, while others have failed to do so [16–19]. Leptin, a polypeptide hormone, functions as an adipostatin, where it suppresses food intake and activates catabolic pathways associated with increased energy production [20, 21]. It also plays a role in improving the insulin sensitivity of the peripheral tissues and affects beta-cell functions. Leptin signaling is involved in obesity and its cardiovascular complications [22]. It also affects reproductive functions at many levels, where it is shown to inhibit folliculogenesis, influences embryo implantation and endometrial receptivity [23–25]. Since obesity is associated with hyperleptinaemia, the situation in obese PCOS becomes more complex [25, 26]. Ghrelin, another peptide hormone, has an orexigenic effect [27]. It also stimulates growth hormone secretion, regulates glucose metabolism, appetite, body weight, endocrine pancreatic, and ovarian functions [28, 29]. It exhibits a negative correlation with androstenedione, and in obese women with PCOS, it may contribute to modification of factors such as insulin resistance and androgens, hence producing a negative effect on fertility [12]. Ghrelin levels are shown to be lower in PCOS, and this decrease has been associated with the negative correlation shown between body mass index (BMI) and ghrelin [30]. Several studies show contradictory results in the level of ghrelin in PCOS patients, where lower ghrelin levels have been reported in some studies, while others report that there was no change in ghrelin level in PCOS, [4, 5, 31, 32]. An earlier study from our group, on a smaller number of Saudi PCOS patients, failed to show the existence of hypoghrelinaemia [19].

Several metabolic and hormonal dysfunctions exist in patients suffering from PCOS, and these include insulin resistance, glucose intolerance, and dyslipidemia [33, 34]. Hyperinsulinaemia is frequently reported in PCOS and may be involved in anovulation, by causing premature maturation of granulosa cells, inhibition of granulosa cell proliferation and development in the presence of elevated LH levels [35]. Furthermore, some studies have shown that insulin resistance and neuroendocrine dysfunctions play a significant role in the pathogenesis of PCOS [36].

In Saudi females, PCOS occurs frequently, but the exact prevalence is not known. Some recent studies have reported variations in the levels of metabolic and hormonal parameters [19]. However, more extensive studies are warranted, in order to achieve a clearer picture of the disease and to identify further underlying etiological factors contributing to the development of PCOS in the Saudi population.

We designed this study as a case-control study on Saudi PCOS patients in an attempt to determine leptin and ghrelin levels and to investigate the type of relationship that exists between these hormones and anthropometric, metabolic and hormonal parameters. The study was conducted in two stages, where initially (stage 1) the results in the PCOS were compared to the control group and in the stage 2, a comparison was made between the lean and obese PCOS. This paper reports the findings of this study in Saudi Arabia and compares the results with those reported for other populations.

## Methods

### Subject recruitment and type of study

The present investigation was conducted as a prospective, observational, cross-sectional, case-control study at the Department of Obstetrics and Gynecology, Al-Noor Hospital, Makkah, Kingdom of Saudi Arabia. The local Ethical Committee approved the study protocol (Review Board (IRB) at the Umm Al Qura University, Makkah Al Mukaramah, Saudi Arabia (IRB No. 235) and 252 females, who were attending the outpatient Gynecology Clinic, were enrolled in the present investigation, after taking their informed consent.

Of the total females enrolled, 130 patients were diagnosed as suffering from PCOS group according to the Rotterdam Consensus guidelines [4], based on the association of at least two of the three following criteria:

1. Oligo- and/or anovulation; confirmed by luteal progesterone and normal serum FSH levels (normal range: 1.0–10.0 mIU/ml).
2. Clinical and/or biochemical signs of raised androgens; elevated serum androgen levels (total testosterone > 2 nmol/l), and/or androstenedione > 0.15 nmol/l, and/or dehydroepiandrosterone sulphate (DHEAS) > 10 mmol/l); LH to FSH ratio > 2.
3. Ultrasound criterion of PCOS; at least one ovary containing > 12 follicles measuring 2–9 mm in diameter and/or increase in the ovarian volume to at least 10 ml [4, 37].

The control group consisted of 121 normo-ovulatory women with male, tubal or unexplained infertility. They had regular ovulatory cycles (25–35 days), no endocrine abnormalities, no clinical or biochemical signs of raised androgens, and normal ultrasonic ovarian morphology. The control women were matched with PCOS patients for age (± 2 years SD) and body mass index, BMI (± 10%).

Exclusion criteria for all the subjects included pregnancy, hypothyroidism, hyperprolactinemia, Cushing’s syndrome, congenital adrenal hyperplasia, current or previous (within the last 6 months) use of oral contraceptives, glucocorticoids, antiandrogens, ovulation induction agents, antidiabetic and anti-obesity drugs or other hormonal pharmaceuticals. None of the patients was affected by neoplastic, metabolic and cardiovascular disorder or other concurrent medical illness such as diabetes, renal disease, and hepatic disorders. All the subjects were non-smokers and had normal physical activity.

### Anthropometric measurements

For each woman weight and height were measured and the BMI (weight in kg divided by height in m^2^) were calculated. Considering our anthropometric local data on the Saudi female population, patients with a BMI > 27 kg/m^2^ were considered obese, and those with a BMI ≤27 were considered lean. Waist circumference (the narrowest circumference between the lower costal margins and the iliac crest) and hip circumference (the maximum circumference at the level of the femoral trochanters) were also measured in the standing position to calculate the waist-hip ratio (WHR).

### Biochemical measurements

A peripheral venous blood sample was extracted in the morning after overnight fasting. During the early follicular phase (2nd or 3rd day), 5 ml of blood was drawn in plain red-top tubes for serum on which the determination of leptin, cholesterol, triglyceride, HDL-C, LDL-C, insulin levels was accomplished within the next 4 h (max) following the blood withdrawal. Two ml of blood were collected into chilled tubes containing 1.2 mg EDTA and aprotinin (500 KIU/ml; Trasylol; Bayer Corp., Leverkusen, Germany) for total ghrelin levels and 2 ml were drawn in fluoride tubes (gray top) for glucose estimation. All blood samples for each woman were immediately centrifuged, and the serum or plasma was stored at − 80 °C until further analysis.

The basal serum levels of insulin were estimated using the electrochemiluminescence immunoassay “ECLlA” on a Roche Elecsys 1010/2010 and Modular Analytics E170 (Elecsys module) immunoassay analyzers (Roche Diagnostic, Mannheim, Germany). Total ghrelin levels were measured in duplicate using a commercial ghrelin (human)-enzyme immunoassay kit (EIA) from Phoenix Pharmaceuticals, Inc.,(Belmont, CA, USA), with a lower limit of detection of 0.06 ng/ml. Plasma glucose levels were determined by the glucose oxidase method on a Beckman Glucose Analyzer (Fullerton, CA). Lipids were determined on an autoanalyzer for clinical chemistry in the hospital.

Homeostatic model assessment of insulin resistance (HOMA-IR) and insulin secretion were calculated using the following formulas:

$$ \mathrm{Insulin}\kern0.17em \mathrm{resistance}=\frac{\mathrm{FI}\times \mathrm{G}}{22.5}\kern2em \mathrm{Insulin}\kern0.5em \mathrm{secretion}=\frac{20\times \mathrm{FI}}{\mathrm{G}\hbox{-} 3.5} $$

HOMA-IR = Fl X G/ 22.3

HOMA-S = 20 X-FI/G-3.5

Where FI is fasting insulin and G is fasting glucose.

### Statistics

Data was collected and entered into a spreadsheet, to evaluate their organization and inference with proper statistical assays, where all analyses were performed using Statistical Package for the Social Science, version 22 (SPSS; Inc., Chicago, IL, USA). Results were expressed as mean ± SD and compared using ANOVA in a student’s *t*-test or a Wilcoxon-Mann-Whitney *U*-test when appropriate, following a Shapiro-Wilk test for normality. Correlations between ghrelin, leptin, HOMA-IR and HOMA-S levels and the anthropometric measurements, lipid, and hormonal parameters were performed and evaluated using Pearson correlation coefficient (*r*).

Multiple regression analysis was used to evaluate the preferential effect of the different studied variables on ghrelin, leptin, HOMA-IR and HOMA-S levels. A *p*-value < 0.05 was considered statistically significant. Data from this study were also approached to build up a ROC curve analysis for the determination of the best suited predictive marker of PCOS in obese Saudi women.

## Results

Anthropometric data and metabolic profile of patients with PCOS and controls are summarized in Table 1. There were no statistically significant differences in the leptin and ghrelin levels between the two investigated groups. In the PCOS group, the WHR, cholesterol, triglyceride, LDL-C and insulin levels were significantly higher and HDL-C levels were significantly lower in comparison with the control group (*p* < 0.0001). Insulin resistance as indicated by the higher HOMA-IR was significantly more in the PCOS compared to the controls, but the insulin secretion as judged from the value of HOMA-S was not different between the two groups.

**Table 1:** Comparison of anthropometric measurements and metabolic profile of patients with polycystic ovary syndrome (POCS) and normal healthy females

	Group^a^	Mean	SD	p
Age (years)	PCOS	25.61	0.39	0.135
	Control	24.67	0.50	
Body mass index (BMI) (kg/m2)	PCOS	28.66	0.58	0.339
	Control	27.77	0.73	
Weight/height ratio	PCOS	0.84	0.008	< 0.0001
	Control	0.76	0.007	
Cholesterol (mmol/L)	PCOS	4.32	0.07	< 0.0001
	Control	3.64	0.05	
Triglyceride (mmol/L)	PCOS	1.08	0.03	< 0.0001
	Control	0.86	0.03	
HDL-C (mmol/L)	PCOS	1.08	0.02	< 0.0001
	Control	1.27	0.03	
LDL-C (mmol/L)	PCOS	2.44	0.05	< 0.0001
	Control	1.71	0.05	
Leptin (ng/ml)	PCOS	25.56	1. 37	0.833
	Control	26.05	1.84	
Ghrelin (ng/ml)	PCOS	0.42	0.013	0.200
	Control	0.45	0.015	
Insulin (pmol/L)	PCOS	97.98	5.20	< 0.0001
	Control	73. 37	3.48	
Glucose (mmol/L)	PCOS	5.05	0.045	< 0.0001
	Control	4.71	0.045	
HOMA-IR (insulin resistance)	PCOS	3.23	0.18	< 0.0001
	Control	2.28	0.11	
HOMA-S (insulin sensitivity)	PCOS	180.66	10.07	0.770
	Control	184.70	9.32	

Group^a^ = PCOS- 130; Control- 122

*SEM* Standard error of the mean; *p* = < 0.05 is statistically significant

*HOMA* Homeostatic model assessment

The lean and obese PCOS and control were separated, and all the parameter values were recalculated and compared to the PCOS (either lean or obese) patients and controls. The results are presented in Table 2.

**Table 2:** Comparison of the anthropometric measurements and metabolic profile of lean and obese patients with polycystic ovary syndrome (POCS) and lean and obese controls

Parameter	Patient Group	Mean	SD	*p* ^1^	Control Group	Mean	SD	*p* ^2^	*p* ^3^
Age (years)	Lean	26.05	0.63	0.131	Lean	23.95	0.60	0.231	0.018
	Obese	27. 24	0.48		Obese	25.14	0.79		0.028
Body mass index (BMI) (kg/m2)	Lean	22.86	0.20	0.0001	Lean	20.85	0. 24	< 0.0001	< 0.0001
	Obese	33.64	0.62		Obese	34.47	0.72		0.387
Weight/height ratio	Lean	0.79	0.01	0.0001	Lean	0.71	0.006	< 0.0001	< 0.0001
	Obese	0.88	0.01		Obese	0.81	0.007		< 0.0001
Cholesterol (mmol/L)	Lean	3.91	0.06	0.0001	Lean	3.42	0.05	< 0.0001	< 0.0001
	Obese	4.68	0.11		Obese	3.86	0.07		< 0.0001
Triglyceride (mmol/L)	Lean	0.93	0.05	0.0001	Lean	0.68	0.029	< 0.0001	< 0.0001
	Obese	1.21	0.05		Obese	1.04	0.057		0.025
HDL-C (mmol/L)	Lean	1.04	0.04	0.132	Lean	1.44	0.040	< 0.0001	< 0.0001
	Obese	1.11	0.04		Obese	1.12	0.038		0.945
LDL-C (mmol/L)	Lean	2.20	0.04	0.0001	Lean	1.31	0.048	< 0.0001	< 0.0001
	Obese	2.65	0.09		Obese	2.11	0.078		< 0.0001
Leptin	Lean	13.65	0.40	0.0001	Lean	11.70	0.464	< 0.0001	< 0.0001
	Obese	35.78	1.78		Obese	39.94	2.59		0.181
Fasting ghrelin	Lean	0.53	0.02	0.0001	Lean	0.57	0.016	< 0.0001	0.025
	Obese	0.33	0.01		Obese	0.33	0.014		0.767
Fasting insulin (pmol/L)	Lean	86.67	8.20	0.04	Lean	52.57	02.28	< 0.0001	< 0.0001
	Obese	107.68	6.45		Obese	93.51	5. 37		0.094
Fasting Glucose (mmol)	Lean	4.93	0.05	0.014	Lean	4.53	0.051	< 0.0001	< 0.0001
	Obese	5.15	0.07		Obese	4.88	0.066		0.007
HOMA-IR (insulin resistance)	Lean	2.807	0.27	0.034	Lean	1.49	0.070	< 0.0001	< 0.0001
	Obese	3.492	0. 25		Obese	2.88	0.177		0.05
HOMA-S (insulin secretion)	Lean	164.80	16.64	0.146	Lean	161.06	10.62	0.013	0.850
	Obese	194. 25	11.97		Obese	207.19	14.64		0.49

*p*^1^ = significance of the difference between lean and obese PCOS

*p*^2^ = significance of the difference between lean and obese control

*p*^3^ = significance of the difference between lean PCOS vs lean control and obese PCOS vs obese control

*HOMA* Homeostatic model assessment

When the lean PCOS were compared with the obese PCOS, all studied parameters including leptin, ghrelin, and HOMA-IR, except HDL level and HOMA-S showed statistically significant differences. However, this difference was not statistically significant when all PCOS patients were compared with controls. On the other hand, all the parameters were different statistically when the lean control values were compared with the values obtained in the obese controls, as shown in Table 2. The differences were still significant statistically when the values in lean PCOS were compared to the lean control, except for HOMA-S, which did not differ significantly. However, when the values in obese PCOS were compared to the values in obese control, certain parameters lost the significant difference. These included BMI, HDL-C, leptin, ghrelin, insulin and HOMA-S.

Table 2 shows leptin and ghrelin levels in lean and obese patients with PCOS compared to controls. Ghrelin levels were significantly lower, and leptin levels were significantly higher in obese patients with PCOS and obese controls in comparison with lean patients with PCOS and lean controls (*P* < 0.0001 for each). Likewise, ghrelin levels were significantly lower (*P* = 0.0249), and leptin levels were significantly higher (*P* = 0.0019) in lean patients with PCOS in comparison with lean controls. However, there was no statistically significant difference in ghrelin levels (*P* = 0.767) and leptin levels (*P* = 0.181) in obese patients with PCOS and obese controls.

Correlation of leptin levels to the clinical and metabolic profile of patients with PCOS and controls is presented in Table 3. In the PCOS group, leptin levels did not correlate with age and glucose levels, however, there was a significant positive correlation with BMI, WHR, cholesterol, triglyceride, LDL-C and insulin levels and a significant negative correlation with HDL-C and ghrelin levels. In the control group, there was only a significant positive correlation with BMI and insulin levels; and a significant negative correlation with ghrelin levels while other parameters did not correlate with leptin levels. Multiple regression analysis demonstrated that insulin was the primary determinant of leptin level in the PCOS group (*R*^*2*^ = 0.352; *P* = 0.0001).

**Table 3:** Correlation between leptin levels and clinical and metabolic profile of patients with polycystic ovary syndrome (POCS) and healthy controls

Correlation of leptin levels and	PCOS (*n* = 130)		Control (*n* = 122)	
	*r*	*p*	*r*	*p*
Age	0.092	0.2979	0.045	0.6226
Body mass index (BMI)	0.289	0.0009**	0.187	0.0392*
Weight/height ratio	0.213	0.0150*	0.096	0.2929
Cholesterol	0.204	0.0199*	0.043	0.6382
Triglyceride	0.175	0.0464*	0.024	0.7930
HDL-C	−0.202	0.0212*	0.037	0.6858
LDL-C	0.235	0.0071*	0.049	0.5920
Glucose	0.135	0.1257	0.121	0.1843
Insulin	0.382	0.0001**	0.254	0.0048*
Ghrelin	− 0.251	0.0040*	− 0.248	0.0059*
HOMA-IR (insulin resistance)	0.316	0.0001**	0.611	< 0.0001**
HOMA-S	0.175	0.046*	0.326	< 0.0001**

*HOMA* Homeostatic model assessment

*Significant (*p* < 0.05)

**Highly significant (*p* < 0.001)

Correlation of ghrelin levels to the clinical and metabolic profile of patients with PCOS and controls is presented in Table 4. In the PCOS group, ghrelin levels did not correlate with age and glucose levels; however, there was a significant negative correlation with BMI, WHR, cholesterol, triglyceride, LDL-C, insulin, leptin levels, HOMA-IR and HOMA-S; and a significant positive correlation with HDL-C levels. In the control group, only a significant negative correlation with BMI, insulin, leptin levels and HOMA-IR and HOMA-S was observed, while other parameters did not correlate with ghrelin levels. Multiple regression analysis demonstrated that insulin was the primary determinant of ghrelin level in the PCOS group (*R*^*2*^ = 0.316; *P* < 0.0001).

**Table 4:** Correlation between ghrelin levels and clinical and metabolic profile of patients with polycystic ovary syndrome (POCS) and healthy controls

Correlation of ghrelin levels and	PCOS (*n* = 130)		Control (*n* = 122)	
	*r*	*p*	*r*	*p*
Age	0.108	0.2213	0.118	0.1955
Body mass index (BMI)	−0.292	0.0007**	− 0.196	0.0305*
Weight/height ratio	−0.209	0.0170*	0.114	0.2112
Cholesterol	−0.215	0.0140*	0.048	0.5996
Triglyceride	−0.199	0.0232*	0.019	0.8354
HDL-C	0.207	0.0181*	0.021	0.8184
LDL-C	−0.229	0.0088*	0.071	0.4371
Glucose	0.129	0.1435	0.109	0.2320
Insulin	−0.347	0.0001**	− 0.251	0.0053*
Leptin	−0.251	0.0040*	−0.248	0.0059*
HOMA-IR (insulin resistance)	−0.279	0.001**	− 0.637	< 0.0001**
HOMA-S (insulin secretion)	− 0.293	0.001**	− 0.257	0.004**

*HOMA* Homeostatic model assessment

*Significant (*p* < 0.05)

**Highly significant (*p* < 0.001)

The HOMA-IR and HOMA-S were correlated with the clinical and biochemical parameters, and the results are presented in Tables 5 and 6, respectively.

**Table 5:** Correlation of homeostatic model assessment for insulin resistance (HOMA-IR) and the clinical and metabolic profile of patients with polycystic ovary syndrome (POCS) and healthy controls

Correlation between HOMA-IR and:	PCOS		Control	
	*r*	*p*	*r*	*p*
Age	−0.183	0.038^*^	0.144	0.115
Body mass index (BMI)	0.342	0.0001^**^	0.634	< 0.0001^**^
Weight/height ratio	0.212	0.024^*^	0.518	< 0.0001^**^
Cholesterol	0.168	0.057	0.382	< 0.0001^**^
Triglyceride	0.325	0.0001^**^	0.350	< 0.0001^**^
HDL-C	−0.210	0.016^*^	−0.389	< 0.0001^**^
LDL-C	0.268	0.002^**^	0.529	< 0.0001^**^
Leptin	0.316	0.0001^**^	0.611	< 0.0001^**^
fasting ghrelin	−0.279	0.001^**^	−0.637	< 0.0001^**^
Fasting insulin	0.970	0.0001^**^	0.984	< 0.0001^**^
Fasting glucose	0.463	0.0001^**^	0.560	< 0.0001^**^
HOMA-S (insulin secretion)	0.698	0.0001^**^	0.405	< 0.0001^**^

*Significant (*p* < 0.05)

** Highly Significant (*p* < 0.001)

**Table 6:** Correlation of homeostasis model assessment of insulin sensitivity (HOMA-S) and the clinical and metabolic profile of patients with polycystic ovary syndrome (POCS) and healthy controls

Correlation between HOMA-S and:	PCOS		Control	
	*r*	*p*	*r*	*p*
Age	−0.320	< 0.0001^**^	0.074	0.420
Body mass index (BMI)	0.194	0.027^*^	0.317	< 0.0001^**^
Weight/height ratio	0.180	0.056	0.343	< 0.0001^**^
Cholesterol	0.079	0.370	0.145	0.111
Triglyceride	0.185	0.035^*^	0.153	0.095
HDL-C	−0.265	0.002^**^	−0.118	0.199
LDL-C	0.175	0.047^*^	0.168	0.065
Leptin	0.175	0.046^*^	0.326	< 0.0001^**^
Fasting ghrelin	−0.293	0.001^**^	−0.257	0.004^**^
Fasting insulin	0.832	< 0.0001^**^	0.540	< 0.0001^**^
Fasting glucose	−0.210	0.017^*^	−0.415	< 0.0001^**^
HOMA-IR (insulin resistance)	0.698	< 0.0001^**^	0.405	< 0.0001^**^

*Significant (*p* < 0.05)

** Highly Significant (*p* < 0.001)

ROC analysis of PCOS patients either without or with obesity diagnosis (lean) for all the parameters investigated and the relative values are shown respectively in Additional file 1: Figures S1, S2 and S3 and Additional file 2: Tables S1, S2 and S3.

## Discussion

The results of this study showed that the PCOS group had significantly higher levels of insulin, insulin resistance, glucose, all plasma lipids except HDL-C, compared to the healthy controls, while insulin secretion was not different. Among the anthropometric variables, the two groups were very similar and only differed in the WHR ratio, which was significantly higher in the PCOS patients.

The abnormalities in lipid, insulin, and HOMA-IR, seen in this study confirm previous reports, which show that PCOS patients have altered lipidograms with high LDL-C, most probably due to the insulin resistance phenotype and altered BMI, rather than circulating androgen levels [38]. This evidence might recall some recent data on Brazilian adolescent women, where the existence of more than one risk factor for type 2 diabetes mellitus showed a high HOMA-IR and a low HOMA-S [39].

Both leptin and ghrelin did not show any differences between the two groups. Hence, our findings could not confirm the earlier reports by Mitkov et al. [17] and Pehlivanov et al. [18] and other researchers [19, 20, 22], who reported an elevated level of leptin in PCOS patients. As a matter of fact, controversial results have been reported for serum leptin levels in PCOS patients, where both high concentrations and unchanged levels have been documented in different studies, though novel parameters, such as the ratio adiponectin/leptin, appear to be more promising to associate PCOS with serum circulating adipokines [4, 40, 41]. These controversial results have been explained by differences in age, anthropometric differences, genetics and disease severity [42, 43].

Leptin has a dual effect on reproduction, where elevated levels of this hormone may have a pathophysiological role in the development of PCOS. A positive effect of leptin is in its role as a trigger of puberty on a hypothalamic-pituitary axis by stimulating estrogen secretion, and the negative impact of leptin, in conditions such as hyperleptinemia, is the inhibition of the ovarian response to gonadotrophin stimulation [44].

When the obese PCOS patients were separated from the lean PCOS patients, and the results were compared with the obese and lean controls, respectively, the lean PCOS had all parameters significantly different compared to the lean controls except for HOMA-S. On the other hand, when the obese PCOS were compared to the obese control, the significant differences were lost between the two groups in BMI, leptin, ghrelin, insulin, HDL-C and HOMA-S, but the differences persisted in all the lipids, in glucose level and insulin resistance.

These results demonstrate that PCOS is associated with hyperinsulinemia, insulin resistance and elevated glucose and lipids, but as the BMI increases the differences in leptin, ghrelin, insulin, HDL-C, and insulin secretion are lost, indicating that BMI, plays a major role in relating to these abnormalities even in the control group.

Several interesting correlations were identified when ghrelin, leptin, HOMA-IR and HOMA-S levels were correlated with the biochemical and hormonal parameters in the PCOS patients and controls. The association between leptin and the lipids were significant in the PCOS, but not in the controls. These results indicate very clearly that even though leptin levels do not differ between the PCOS and controls, but the leptin levels significantly correlate with elevating lipids, insulin, ghrelin, insulin resistance and insulin secretion. The findings of the present study confirm the result of previous studies, which have shown a significant correlation between WHR with leptin in the PCOS patients [45, 46]. This supports the importance of abdominal fat mass in the secretion of leptin and hence in the resulting lipid abnormalities which exist in the PCOS. Ghrelin correlated with the same parameters as leptin, except that the correlations were inverse. The WHR was significantly higher in the PCOS group, both lean and obese group compared to the counterpart control groups. These results further confirm the importance of the abdominal fat in PCOS.

Insulin resistance is a frequent finding in PCOS and the PCOS patients in this study had higher levels of HOMA-IR compared to the control group. Correlation studies between HOMA-IR and the clinical and biochemical parameters showed several correlations in both patients and controls, except between HOMA-IR and cholesterol in the patient group, there was no relationship. These results indicate that insulin resistance plays a significant role in the associated hormonal and biochemical abnormalities observed in PCOS patients [10, 47–49].

Insulin secretion as judged from the level of HOMA-S did not differ between the PCOS patients and controls (*p* > 0.05), and comparison between PCOS lean and obese females did not show any significance (Table 2).

This study is important as it has highlighted several differences in the results of Saudi PCOS patients compared to studies reported in literature. It has also shown significant differences between the obese and lean PCOS patients. Though this study has covered several parameters, the major limitation of this study is that coagulation parameters and liver functions were not investigated, and hence the findings of previous studies, related to these parameters, could not be confirmed in Saudis.

Further studies are warranted on a larger group of PCOS patients in an attempt to confirm the role of liver disorders and coagulation parameter abnormalities in the pathogenesis of PCOS in Saudi patients.

## Conclusion

This study investigated the relationship between leptin and ghrelin in PCOS-affected Saudi women and showed that leptin is probably associated with lipidograms (particularly with chol-LDL) in PCOS patients, as expected, but that the difference in serum adipokines levels between lean and obese female with PCOS, is no more significant, most probably due to an interference of insulin level and HOMA indexes [50–58]. It may be concluded that leptin or ghrelin levels in the serum are associated with insulin metabolism and insulin resistance and the lipidogram pattern and BMI is a consequence of the insulin metabolic impairment. This evidence is supported by the interesting data from HOMA-S.

## Additional files

Additional file 1: **Figure S1.** ROC-Curve of all the investigated parameters in PCOS patients. **Figure S2.** ROC-Curve of all the investigated parameters in lean PCOS patients. **Figure S3.** ROC-Curve of all the investigated parameters in Obese PCOS patients. (DOCX 403 kb)

Additional file 2: **Table S1.** ROC-Curve of all the investigated parameters in PCOS-Lean patients. **Table S2.** ROC-Curve of all the investigated parameters in PCOS-Obese patients. **Table S3.** ROC-Curve of all the investigated parameters in all PCOS patients. (DOCX 20 kb)

## Abbreviations

BMI: Body Mass Index
DHEAS ESHR and E/ASRM: Embryology/American Society for Reproductive Medicine
ECLlA: electrochemiluminescence immunoassay
FI: Fasting Insulin
G: Glucose
HDL: high-density lipoprotein
HOMA-IR: Homeostatic model assessment of insulin resistance
HOMA-S: Homeostatic model assessment of insulin secretion
LDL: low-density lipoprotein
PCOS: polycystic ovary syndrome
ROC: Receiver Operator characteristic curve
SEM: Standard error of the mean
