# Supplementary material for: A study of ghrelin and leptin levels and their relationship to metabolic profiles in obese and lean Saudi women with polycystic ovary syndrome (PCOS)
Source: Lipids Health Dis. 2018 Aug 21;17:195. doi: 10.1186/s12944-018-0839-9 (PMC6103980; doi:10.1186/s12944-018-0839-9)
Supplement: Supplementary file 1 — Figure S1. ROC-Curve of all the investigated parameters in PCOS patients. Figure S2. ROC-Curve of all the investigated parameters in lean PCOS patients. Figure S3. ROC-Curve of all the investigated parameters in Obese PCOS patients. (DOCX 403 kb) [file 12944_2018_839_MOESM1_ESM.docx]

Additional file 1

Figures 1S: ROC-Curve of all the investigated parameters in PCOS patients

Figures 2S: ROC-Curve of all the investigated parameters in lean PCOS patients

Figures 3S: ROC-Curve of all the investigated parameters in Obese PCOS patients

Figures 1S: ROC-Curve of all the investigated parameters in PCOS patients

Figures 2S: ROC-Curve of all the investigated parameters in lean PCOS patients

Figures 3S: ROC-Curve of all the investigated parameters in obese PCOS patients
