# Supplementary material for: A study of ghrelin and leptin levels and their relationship to metabolic profiles in obese and lean Saudi women with polycystic ovary syndrome (PCOS)
Source: Lipids Health Dis. 2018 Aug 21;17:195. doi: 10.1186/s12944-018-0839-9 (PMC6103980; doi:10.1186/s12944-018-0839-9)
Supplement: Supplementary file 2 — Table S1. ROC-Curve of all the investigated parameters in PCOS-Lean patients. Table S2. ROC-Curve of all the investigated parameters in PCOS-Obese patients. Table S3. ROC-Curve of all the investigated parameters in all PCOS patients. (DOCX 20 kb) [file 12944_2018_839_MOESM2_ESM.docx]

Additional file 2

**Table S1**. ROC-Curve of all the investigated parameters in PCOS-Lean patients

| Parameters | Area under the curve | Cut-off value | Sensitivity % | Specificity % | P value |
| --- | --- | --- | --- | --- | --- |
| Age (years) | 0.630 | 26.500 | 50.0 % | 78.3 % | 0.014 |
| BMI (kg/m2) | 0.792 | 20.200 | 95.0 % | 53.3 % | <0.0001 |
| Waist | 0.815 | 72.500 | 65.0 % | 86.7 % | <0.0001 |
| Hip | 0.619 | 91.750 | 85.0 % | 41.7 % | 0.025 |
| W/H ratio | 0.813 | 0.725 | 85.0 % | 66.7 % | <0.0001 |
| Cholesterol (mmol/L) | 0.792 | 3.750 | 85.0 % | 80.0 % | <0.0001 |
| Triglyceride (mmol/L) | 0.750 | 0.660 | 90.0 % | 56.7 % | <0.0001 |
| HDL (mmol/L) | 0.812 | 1.405 | 100.0 % | 46.7 % | <0.0001 |
| LDL (mmol/L) | 0.958 | 1.850 | 90.0 % | 91.7 % | <0.0001 |
| Leptin (ng/ml) | 0.667 | 11.750 | 80.0 % | 48.3 % | 0.002 |
| Fasting Ghrelin | 0.627 | 0.505 | 65.0 % | 58.3 % | 0.017 |
| Fasting Insulin (pmol/L) | 0.624 | 73.750 | 40.0 % | 88.3 % | 0.019 |
| Fasting Glucose | 0.781 | 4.550 | 95.0 % | 55.0 % | <0.0001 |

**Table S2**. ROC-Curve of all the investigated parameters in PCOS-Obese patients

| Parameters | Area under the curve | Cut-off value | Sensitivity % | Specificity % | P value |
| --- | --- | --- | --- | --- | --- |
| Age (years) | 0.639 | 22.500 | 84.3 % | 53.2 % | 0.006 |
| BMI (kg/m2) | 0.563 | 30.550 | 44.3 % | 71.0 % | 0.214 |
| Waist | 0.527 | 88.500 | 71.4 % | 40.3 % | 0.594 |
| Hip | 0.656 | 110.500 | 61.4 % | 64.5 % | 0.002 |
| W/H ratio | 0.752 | 0.855 | 55.7 % | 83.9 % | <0.0001 |
| Cholesterol (mmol/L) | 0.763 | 4.850 | 52.9 % | 96.8 % | <0.0001 |
| Triglyceride (mmol/L) | 0.617 | 0.725 | 88.6 % | 32.3 % | 0.021 |
| HDL (mmol/L) | 0.520 | 0.930 | 35.7 % | 80.6 % | 0.690 |
| LDL (mmol/L) | 0.719 | 2.250 | 70.0 % | 66.1 % | <0.0001 |
| Leptin (ng/ml) | 0.533 | 48.000 | 88.6 % | 24.2 % | 0.513 |
| Fasting Ghrelin | 0.517 | 0.275 | 74.3 % | 32.3 % | 0.731 |
| Fasting Insulin (pmol/L) | 0.560 | 114.000 | 48.6 % | 66.1 % | 0.233 |
| Fasting Glucose | 0.614 | 4.650 | 87.1 % | 32.3 % | 0.025 |

**Table S3**. ROC-Curve of all the investigated parameters in all PCOS patients

| Parameters | Area under the curve | Cut-off value | Sensitivity % | Specificity % | P value |
| --- | --- | --- | --- | --- | --- |
| Age (years) | 0.641 | 24.500 | 68.5 % | 59.8 % | <0.0001 |
| BMI (kg/m2) | 0.564 | 20.200 | 97.7 % | 26.2 % | 0.078 |
| Waist | 0.623 | 76.500 | 78.5 % | 50.8 % | 0.001 |
| Hip | 0.503 | 121.500 | 93.1 % | 18.9 % | 0.931 |
| W/H ratio | 0.735 | 0.825 | 56.9 % | 81.1 % | <0.0001 |
| Cholesterol (mmol/L) | 0.746 | 3.750 | 85.4 % | 59.0 % | <0.0001 |
| Triglyceride (mmol/L) | 0.677 | 0.660 | 92.3 % | 41.0 % | <0.0001 |
| HDL (mmol/L) | 0.661 | 1.015 | 53.1 % | 73.8 % | <0.0001 |
| LDL (mmol/L) | 0.795 | 1.950 | 83.8 % | 68.0 % | <0.0001 |
| Leptin (ng/ml) | 0.545 | 11.750 | 90.8 % | 23.8 % | 0.219 |
| Fasting Ghrelin | 0.548 | 0.405 | 56.2 % | 57.4 % | 0.184 |
| Fasting Insulin (pmol/L) | 0.609 | 87.450 | 48.5 % | 74.6 % | 0.003 |
| Fasting Glucose | 0.692 | 4.550 | 92.3 % | 40.2 % | <0.0001 |
